# A New Classification of the Anatomical Variations of Labbé’s Inferior Anastomotic Vein

**DOI:** 10.3390/tomography8050183

**Published:** 2022-08-30

**Authors:** Dragoş Ionuţ Mincă, Mugurel Constantin Rusu, Petrinel Mugurel Rădoi, Sorin Hostiuc, Corneliu Toader

**Affiliations:** 1 Division of Anatomy, Department 1, Faculty of Dental Medicine, “Carol Davila” University of Medicine and Pharmacy, RO-020021 Bucharest, Romania; 2 Division of Neurosurgery, Department 6—Clinical Neurosciences, Faculty of Medicine, “Carol Davila” University of Medicine and Pharmacy, RO-020021 Bucharest, Romania; 3 Clinic of Neurosurgery, “Dr. Bagdasar-Arseni” Emergency Clinical Hospital, RO-041915 Bucharest, Romania; 4 Department of Legal Medicine and Bioethics, Faculty of Dental Medicine, “Carol Davila” University of Medicine and Pharmacy, RO-020021 Bucharest, Romania

**Keywords:** vein of Labbé, cerebral vein, tentorium cerebelli, tentorial sinus, superficial middle cerebral vein, computed tomography

## Abstract

(1) Background: The inferior anastomotic vein of Labbé (LV) courses on the temporal lobe, from the sylvian fissure towards the tentorium cerebelli and finishes at the transverse sinus (TS). The importance of the LV topography is related to skull base neurosurgical approaches. Based on the hypothesis of the existence of as yet unidentified anatomical possibilities of the LV, we aimed through this research to document the superficial venous topographic patterns at the lateral and inferior surfaces of the temporal lobe. (2) Methods: A retrospective cohort of 50 computed tomography angiograms (CTAs) of 32 males and 18 females was documented. (3) Results: Absent (type 0) LVs were found in 6% of cases. Anterior (temporal, squamosal–petrosal–mastoid, type 1) LVs were found in 12% of cases. LVs with a posterior, temporoparietal course (type 2) were found to be bilateral in 46% of cases and unilateral in 36% of cases. Type 3 LVs (posterior, parietooccipital) were found to be bilateral in 8% and unilateral in 32% of cases. In 24% of cases, duplicate LVs were found that were either complete or incomplete. A quadruplicate LV was found in a male case. On 78 sides, the LV drained either into a tentorial sinus or into the TS. (4) Conclusions: The anatomy of the vein of Labbé is variable in terms of its course, the number of veins and the modality of drainage; thus, it should determine personalized neurosurgical and interventional approaches. A new classification of the anatomical variations of Labbé’s vein, as detected on the CTAs, is proposed here (types 0–3).

## 1. Introduction

Temporal lobe venous drainage is important in various neurosurgical procedures and combined skull base approaches [1]. The most important draining vein of the temporal lobe is the inferior anastomotic vein (Labbé’s vein, LV) [1]. During fetal development, the LV, an anastomosis between the middle and inferior cerebral veins, is identifiable at 20 weeks; the superior anastomotic vein (vein of Trolard), which connects the superior and middle cerebral veins, appears after 30 weeks [2].

The superficial middle cerebral vein, or superficial sylvian vein (SV), drains most of the lateral surface of the cerebral hemisphere and follows the lateral (sylvian) fissure to terminate in the cavernous sinus [2]. The superior anastomotic vein (vein of Trolard) passes between the SV and the superior sagittal sinus, thus, connecting it with the cavernous sinus [2]. Commonly, the veins of Trolard and Labbé are alternatively present, thus, dominant. The LV courses the temporal lobe and connects the SV with the transverse sinus (TS) [2]. Currently, the term LV is used to indicate the largest vein in the lateral aspect of the temporal lobe [3]. It could be regarded as a bridging vein that courses on the temporal lobe between the sylvian fissure and the TS [4]. The intratentorial course of the LV is regarded as a tentorial sinus (TeS) [5]. According to Koperna (1992), the LV can drain in the TS either via a TeS or via a dural lacuna in the external cranial wall, placed superior to the TS [5]. However, Tubbs (2012) identified only the direct drainage of the LV in the TS [6].

Little attention has been paid to superficial anastomotic veins in studies, although severe postoperative complications may be caused by an iatrogenic venous injury during intracranial surgery [7,8]. The importance of LV topography is related to skull base approaches and medial tentorial lesions, resulting in posterior temporal approaches [7]. Clinicians, students and teachers should pay attention to individual anatomical characteristics, including different manifestations of variability [9]. Understanding the anatomy of the anastomotic veins is of major importance for neurosurgical procedures [10].

Based on the hypothesis of the existence of as yet unidentified anatomical possibilities of the LV, through this research, in a batch of retrospective computed tomography angiograms (CTAs), we aimed to document the superficial venous topographic patterns at the lateral and inferior surfaces of the temporal lobe.

## 2. Materials and Methods

We studied a retrospective randomized cohort of 50 CTAs. Of these, 32 were in male cases and 18 in female cases. The inclusion criteria were the age of the subjects (>18 years), adequate quality of the CTAs and no pathologic processes distorting the vascular anatomy. The exclusion criteria were pathological processes distorting the vascular anatomy and degraded or incomplete CTAs.

The research was conducted following the principles of the World Medical Association Code of Ethics (Declaration of Helsinki). All subjects gave their informed consent for inclusion before they participated in the study. The responsible authorities (affiliation 3) approved the study (approval no. 2093/1 March 2022).

The CT scans were performed with a 32-slice scanner (Siemens Multislice Perspective Scanner), with a 0.6 mm collimation and a reconstruction of 0.75 mm thickness with a 50% overlap for a multiplanar maximum intensity projection and a three-dimensional volume rendering technique, as described previously [11]. The cases were documented using Horos for iOS (Horos Project).

To evaluate the number of cases of each variant, we used frequencies. To evaluate significant associations between qualitative variables, we used the Pearson Chi2 test. We used SPSS v28 for Mac to perform statistical analyses.

We defined four topographic types of LV: type 0—absent LV; type 1—anterior temporal type, with the LV having a course mapping only components of the temporal bone (squamosal–petrosal–mastoid type); type 2—an intermediate, temporoparietal type, with the LV having a course corresponding to the temporal squama (optional), the mastoid angle of the parietal and the mastoid portion of the temporal (squamosal–parietomastoid type); and type 3—posterior parietooccipital type, with the LV having a course corresponding to the temporal squama (optional), the mastoid angle of the parietal and the occipital squama (squamosal–parieto-occipital type). There were situations with multiple (duplicate, triplicate or quadruplicate) LVs recorded. We also recorded the mode of LV drainage into the TS: (type A) via a TeS, (type B) directly or (type C) via a dural lacuna.

## 3. Results

The distribution of genders was homogenous for each variant, with no association found to be statistically significant (see Table 1).

Type 0, an absent LV, was identified as follows: in 3/50 cases (6%), two male and one female, the LV was absent bilaterally. Type 0 was identified unilaterally on the right side in five specimens, four male and one female. Unilateral type 0 was evident on the left side in five different cases, two male and three female.

Type 1, a temporal LV, was not found bilaterally in any case in the documented batch. It was found unilateral in 6/50 cases (12%), with equal left–right prevalence (3 cases on each side); on the right side in 2 male cases and 1 female case, and on the left side in 2 female cases and 1 male case.

**Figure 1:**
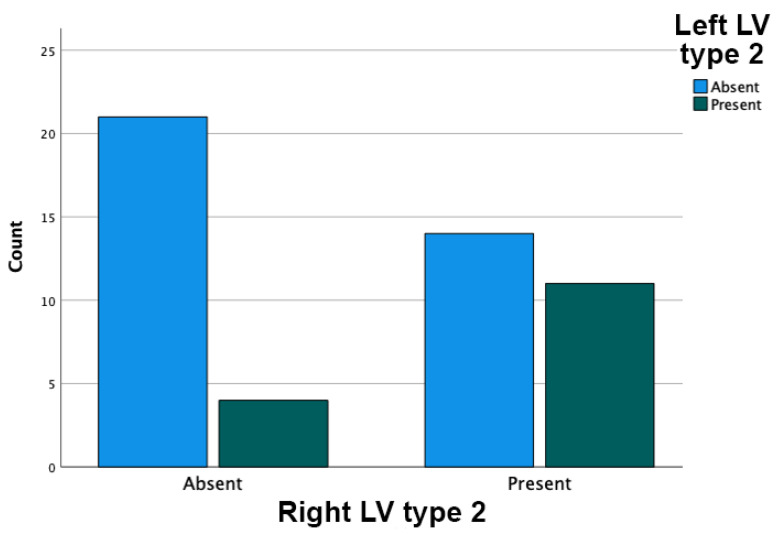
Right and left Labbé’s vein (LV) type 2 association.

Type 2, intermediate or temporoparietal, was present bilaterally in 23/50 cases (46%) and unilaterally in 18/50 cases (36%). Unilateral presence was found in 14/50 cases on the right side and in 4/50 cases on the left side. The presence of a right LV type 2 was significantly associated with the presence of a left LV type 2 (Pearson Chi2 = 4.667; *p* = 0.031; see Figure 1). Briefly, the bilateral symmetry of the LV type 2 was statistically significant.

Type 3, posterior or parieto-occipital, was present bilaterally in 4/50 cases (8%), one male and three female. This type was present unilaterally in 16/50 cases (32%), on the right side in 4 cases, 2 male and 2 female, and on the left side in 12 cases, 10 male and 2 female.

**Figure 2:**
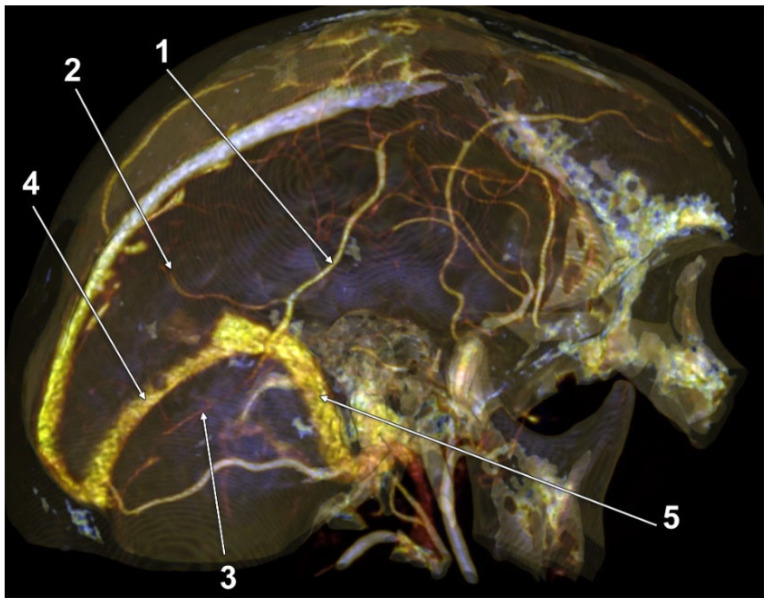
Three-dimensional volume rendering, superomedial endocranial view, left side. Labbé’s vein was not emptying into the transverse sinus. 1. Labbé’s vein; 2. Dural vein; 3. Tentorial vein; 4. Transverse sinus; 5. Sigmoid sinus.

In a male case, we found a topographic type 2 of the left LV. Interestingly, it was not draining into the TS, but had a pathway across it contiguous with branches in the occipital dura mater and tentorium (Figure 2).

**Figure 3:**
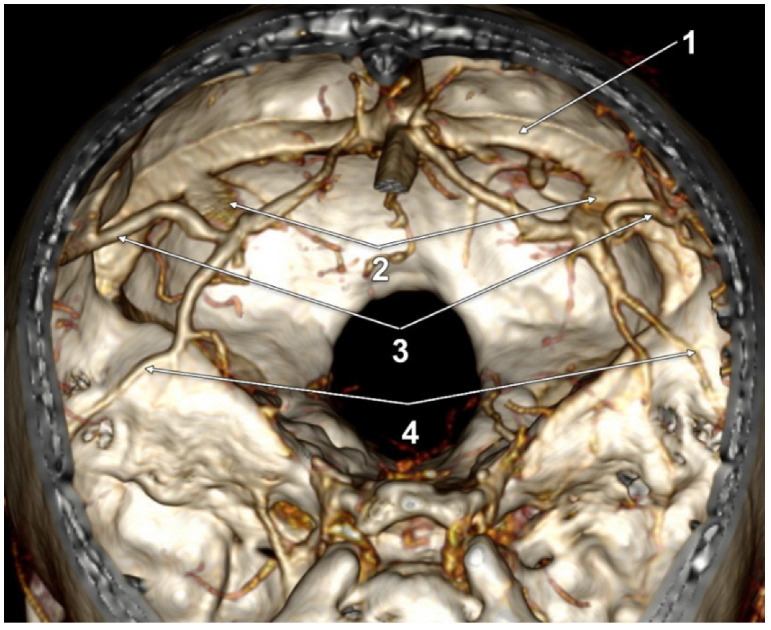
Three-dimensional volume rendering, endocranial anterosuperior view. Bilateral duplicated Labbé’s veins. 1. Left transverse sinus; 2. Tentorial sinuses; 3. Labbé’s vein type 3 (posterior, parieto-occipital), draining into tentorial sinuses; 4. Labbé’s vein type 1 (anterior, temporal), draining into tentorial sinuses but continuing and terminating in the confluence of dural sinuses.

**Figure 4:**
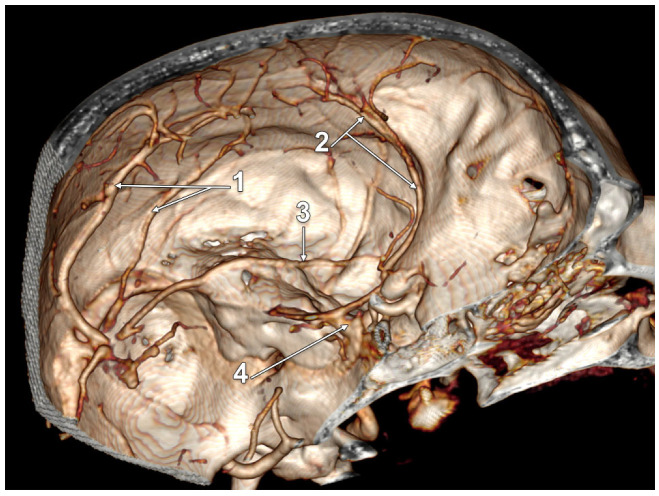
Three-dimensional volume rendering, postero-supero-medial endocranial view, left side. Complete duplication of Labbé’s vein. Sinus of the lesser sphenoidal wing. 1. Complete duplication of Labbé’s vein, combination of types 2 and 3; 2. Lesser wing sinus (sphenoidal segment of the sinus of Breschet); 3. Ophtalmopetrosal sinus of Hyrtl; 4. Cavernous sinus.

**Figure 5:**
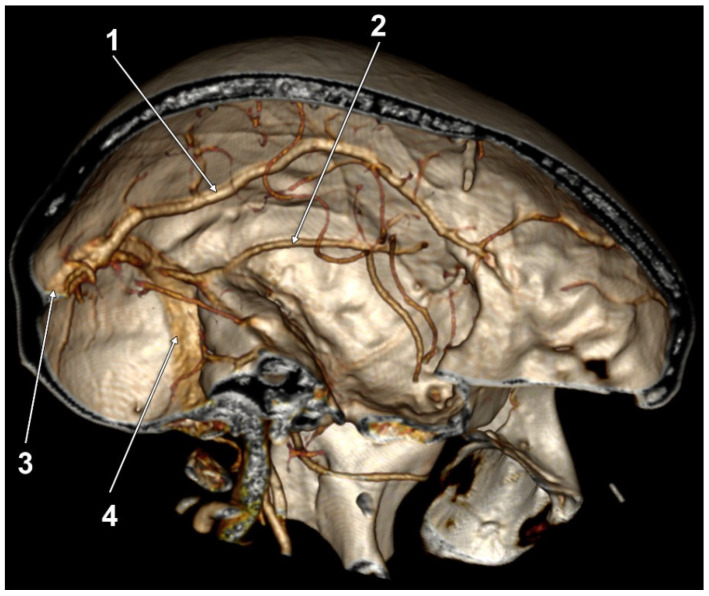
Three-dimensional volume rendering, medial endocranial view, left side. Complete duplication of Labbé’s vein, combination of types 2 and 3. The posterior component appeared topographically as a sagittal parietooccipital vein. 1. Posterosuperior vein of Labbé (type 3); 2. Anteroinferior vein of Labbé (type 2); 3. Transverse sinus; 4. Sigmoid sinus.

**Figure 6:**
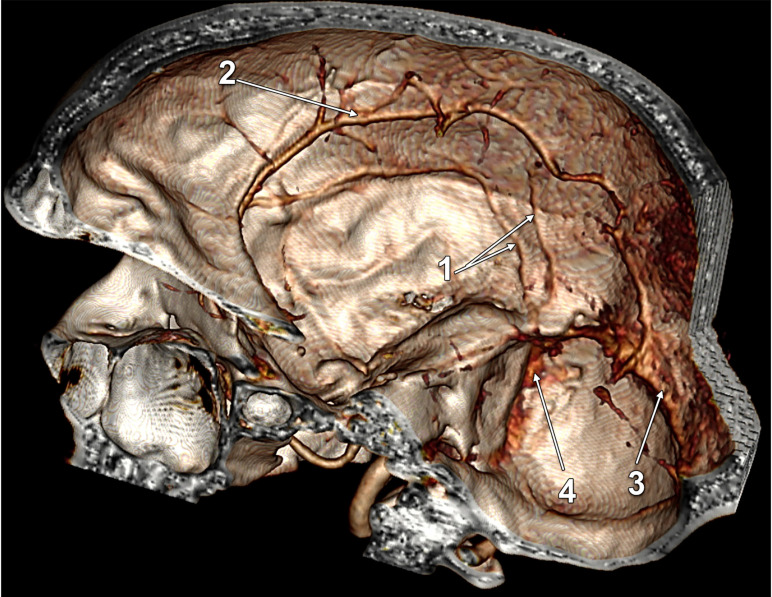
Three-dimensional volume rendering, anteromedial endocranial view, right side. Partial or incomplete duplication of Labbé’s vein. 1. Labbé’s vein partially duplicated; 2. Sphenoparietal sinus of Breschet resulting from anatomical connection of a sagittal parietal vein to the sinus of the lesser sphenoidal wing; 3. Transverse sinus; 4. Sigmoid sinus.

**Figure 7:**
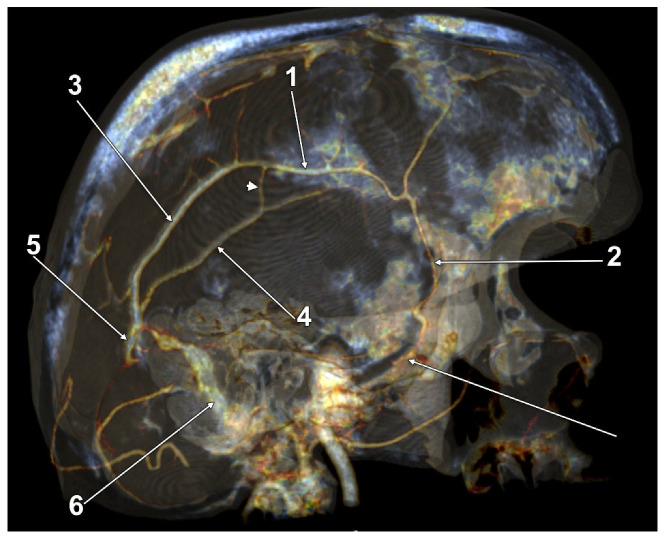
Three-dimensional volume rendering, postero-supero-medial endocranial view, left side. Partial duplication of Labbé’s vein (types 3 + 3). 1. Sagittal parietal vein; 2. Lesser wing sinus; 3. Posterior duplicate of Labbé’s vein; 4. Anterior duplicate of Labbé’s vein, anastomosed with the sagittal parietal vein (arrowhead); 5. Transverse sinus; 6. Sigmoid sinus.

**Figure 8:**
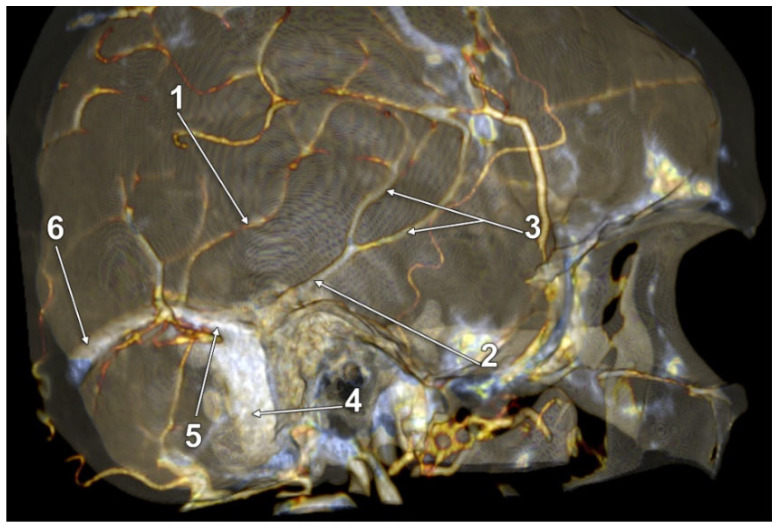
Three-dimensional volume rendering, medial endocranial view, left side. Complete duplication of Labbé’s vein, anterior component shows another duplication, albeit partial. 1. Posterior Labbé’s vein, type 3; 2. Anterior Labbé’s vein, type 2; 3. Proximal duplication of anterior Labbé’s vein; 4. Sigmoid sinus; 5. Tentorial sinus; 6. Transverse sinus.

In 12/50 cases (24%), duplicate LVs were found in 7 male and 5 female cases. Of these, one male case had a complete bilateral LV duplication (2%); its morphology had bilateral symmetry, consisting of a combination of types 1 + 3: (a) an anterior LV, type 1 temporal, draining into a wide TeS, but continuing to terminate in the confluence of sinuses; and (b) a posterior LV, type 3, posterior or parieto-occipital, draining convergently with the anterior one only into the TeS on that side (Figure 3). We found unilateral duplications in 11 cases, 2 on the right side and 9 on the left side. A right duplicated LV was present only in male cases. A left duplicated LV was identified in four male and five female cases. Unilateral duplication was either complete (Figure 4 and Figure 5), with distinct terminal drainage or incomplete (Figure 6 and Figure 7), with terminal drainage through a common trunk (Table 2). One female case had a complete type 2 + 3 duplication of the left LV, but the anterior type 2 component also had a secondary partial duplication (Figure 8).

**Figure 9:**
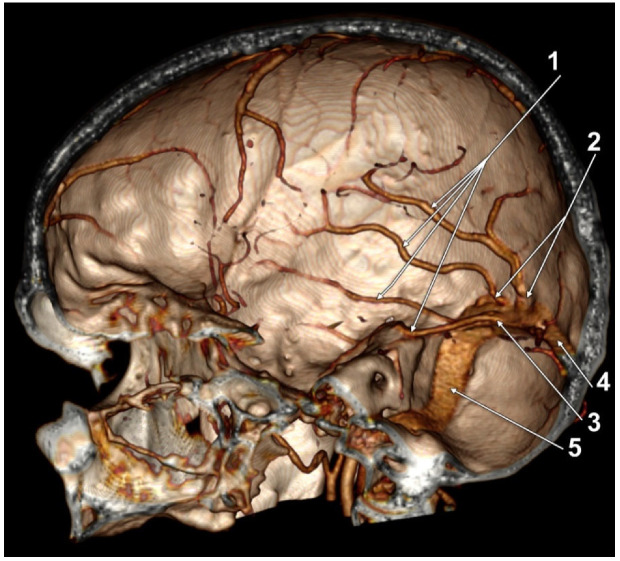
Three-dimensional volume rendering, antero-medial endocranial view, right side. Quadruplicated Labbé’s vein. 1. Palisade of four veins corresponding to the course of Labbé’s vein; 2. Dural lacunae; 3. Tentorial sinus; 4. Transverse sinus; 5. Sigmoid sinus.

In a male case, we identified on the right side a quadruplicated LV (Figure 9). The four veins descending laterally and then inferiorly from the temporal lobe formed a palisade from anteroinferior to posterosuperior, corresponding in this sequence to the 1-2-2-3 LV types defined in this study. The first two connected distally to a TeS, through which they drained into the TS. The latter two drained through the dural lacunae also into the TS.

After separating the cases into those with duplicated and quadruplicated LVs, as well as the 16 sides with absent LVs (type 0), we recorded the drainage modality for 100 LVs. The drainage was: (i) type A in the TS for 49 LVs, 25 right and 24 left; (ii) type B in the TS for 29 LVs, 13 right and 16 left; (iii) type C in the TS for 21 LVs, 11 right and 10 left; (iv) and in one case (Figure 2), the left LV did not drain into the TS, but dichotomized distally into the tentorial and dural occipital veins.

## 4. Discussion

The intracranial venous channels undergo major changes during embryonic development before reaching adult anatomy [12]. The primitive, or embryonic, tentorial sinus (ETS) is the embryonic dural sinus that drains the telencephalic vein, the future superficial middle cerebral vein (SV) [12]. The ETS initially drains into the primitive marginal sinus at the level of the fetal telencephalon, forming the superior sagittal sinus and TS [12]. The redistribution of the venous drainage from the ETS towards the cavernous sinus is called the ‘cavernous sinus capture’; it occurs later in development and determines the drainage pattern of the SV in the adult [12].

The ETS usually regresses postnatally, but various primitive phenotypes may eventually persist (such as sphenoparietal, cavernous, emissary, superior petrosal, basal and squamosal) [13,14]. In these regards, the vein we indicated as the “sagittal parietal vein” bona fide corresponds to the parietal segment of a persisting ETS. When that sagittal parietal vein elongates posteriorly and drains into the TS, it could appear as a posterior duplication of the LV; if it continues anteriorly, it builds up in the sinus of Breschet [15]. When the anatomic variation of the SV was studied [14], the vein of Labbé was overlooked.

The tentorial sinuses have been classified into four groups according to the territory they drained into: group I, in which the sinuses receive blood from the cerebral hemisphere via bridging veins; group II, in which the sinuses are drained and formed by terminal portions of the cerebellar hemispheric or vermian veins; group III, in which numerous veins originating in the tentorium merge, forming a small TeS; and group IV, in which the TeS is formed by a bridging vein from the cerebral hemisphere or brainstem to the tentorial free edge [16]. The preservation and hemostasis of the TeS is important to reduce venous congestion and massive bleeding [17]. Shibao et al. (2019) observed that bridging veins and the TeS have only been reported in cadaveric studies [17]. A case of the TeS draining telencephalic and diencephalic tributaries of the basal vein has been reported [18]. Dorsally, that TeS merged with the straight sinus and, subsequently, with the confluence of sinuses [18]. The authors discussed that attention should be paid to this normal variation whenever a transtentorial surgical approach is considered [18].

A variant of two-point venous drainage was also identified in this study, such as the posterior component of a duplicated LV draining both into a TeS and into the confluence of sinuses. It is interesting to discuss that the TeS was divided into lateral and medial groups, the lateral ones resulting from the convergence of veins from the lateral and basal surfaces of the temporal and occipital lobes [19]. The lateral TeS usually drains into the junction of the TS with the sigmoid sinus, as well as into the anterior two-thirds of the TS [19]. A medial TeS usually drains into the straight sinus and into the confluence of sinuses. For this reason, the outlined variant appeared atypical, because the drainage of the posterior LV was through a medial TeS to the confluence of sinuses, and not through a lateral TeS to the TS.

In type 1, the vein of Labbé courses successively on the temporal bone components: the squama, petrous part and mastoid. It is, therefore, located between the tympanic cavity and the basal surface of the temporal lobe. That course corresponds to the petrosquamous suture and the vein should be anatomically distinguished from the ophtalmopetrosal sinus of Hyrtl and the petrosquamous sinus. The sinus of Hyrtl courses from the superior orbital fissure, successively over the greater sphenoidal wing and the temporal pyramid, emptying either into the superior petrosal sinus or directly into the TS [20,21]. The petrosquamous sinus connects posteriorly to the junction of the superior petrosal and transverse sinuses, but is anteriorly connected either with the retromandibular vein via a postglenoid foramen (spurious jugular foramen) or with the pterygoid plexus via the foramen ovale [21,22]. Therefore, both the sinus of Hyrtl and the petrosquamous sinus are related to the basal surface of the temporal lobe, but not to the lateral one. The proximity of the cerebral cortical veins to the overlying dura predisposes them to surgical injury; therefore, LV interruption during otologic surgery may produce devastating neurologic consequences [23]. Approaches nearing the tegmen tympani should, therefore, take care not to damage any of the sinus of Hyrtl, the petrosquamous sinus or the LV, especially if it is temporal, type 1.

In this study, we found that the type 2 of the LV, which is the classical textbook type, significantly occurred bilaterally. However, the occurrence of the type 1 was <50%. This indicated that types 1 and 3 of the LV we defined were rather atypical variants that could be equally encountered.

LV duplication was found in 31% of cases and appeared to occur predominantly on the left side [7], which was also supported by the results of this study. The LV is rarely absent or poorly developed [3,7]. Mainly, the drainage point of the LV in the TS is located midway between the inion and the external auditory meatus [7]. LV dominance was detected in 31% of cases, more frequently on the left side, by Silva et al. [7]. Shoman et al. (2011) found dominant LVs in 20% of cases, mostly on the right side [3].

During combined craniotomies of the middle and posterior cranial fossae (“petrous” approaches), the LV, which connects the basal face of the temporal lobe to the TS, could be compromised [8]. The occlusion of the LV, which may be the only venous drainage pathway to a large brain territory of the temporal and parietal lobes, can lead to devastating neurological damage, such as disturbances of speech and memory, cerebral edema and, occasionally, contralateral motor deficits [8,24]. For this reason, the presence of multiple LVs, albeit with contiguous cerebral territories, may represent an anatomical safety feature.

The sacrifice of this vein, especially if unique, in a poorly anastomosed superficial cortical venous system, would lead to a swollen temporal lobe and brainstem compression, as well as a posterior hemorrhagic infarct [10]. A preoperative digital subtraction angiography or computed tomography venography could be of help to design a neurosurgical approach [24]. When approaching the cerebellum, the lowest chance of injury to the LV is with the median approach [24]. On other hand, when the imaging examination reveals that the LV is too close to the confluence of sinuses or the dural veins are too long, the approach should be redesigned [24].

A LV draining via a TeS is more susceptible to damage during tentorial division than LVs with different drainage patterns. Therefore, studies exclusively indicating a direct drainage of the LV into the TS, although rigorously conducted, could mislead the neurosurgeons. Typical tentorial and TS drainage [25], and atypical drainage patterns, such as into occipital dural veins, that we and other authors [1] found, point out that it should be mandatory that the drainage of the LV be documented preoperatively. Moreover, multiple LVs could have different drainage patterns; thus, if a posterior one is avoided, an anterior one could be damaged when transtentorial corridors are accessed.

LV thrombosis is a rare entity [4]. Thrombosis of the LV usually tends to occur along with extensive dural venous sinus thrombosis [26]. There is a strong association between the extent of thrombosis in the TS and the presence of LV thrombosis [4]. Moreover, LV thrombosis can extend further to the sigmoid sinus and internal jugular vein [27]. Interestingly, arachnoid granulations could be found at the junction of the LV and TS in more than 20% of cases, and should be differentiated from LV or TS thrombosis [28].

There are multiple endovascular routes to the cavernous sinus, including through the LV [29]. A carotid-cavernous fistula was approached through the LV–SV pathway [30]. As the drainage pattern of the SV is individually variable and does not mandatorily include the LV [12,13,31,32,33], the latter vein’s anatomy should be documented prior to any endovascular approach at the level of the skull base.

## 5. Conclusions

The anatomy of the vein of Labbé is variable; thus, it should be used to determine personalized neurosurgical and interventional approaches. The vein of Labbé’s course and the number of such veins do not conform to a single pattern. Specific approaches to the tentorium should be performed with caution, as the drainage pattern of the vein of Labbé is individually variable in the posterolateral angle of the tentorium. Tentorial sinuses, if present, should also be evaluated individually, to fit the surgical corridor with their specific topography.

## Figures and Tables

**Table 1:** Associations between gender and topographic types of Labbé’s vein (LV).

Variant/Type	Pearson Chi2 Value	*p* Value (Two-Sided)
Right LV/0	0.5	0.479
Left LV/0	0.81	0.368
Right LV/1	0.01	0.921
Left LV/1	1.303	0.254
Right LV/2	0	1
Left LV/2	2.381	0.123
Right LV/3	2.903	0.088
Left LV/3	0.230	0.631

**Table 2:** Morphological variability of unilateral duplication of Labbé’s vein by gender (M: male; F: female). Combinations of topographic types (1–3). Partial (*p*) or complete (C) duplication.

Gender	Right Side	Left Side
M	2 + 2 *p* (Figure 6)	
M	1 + 3 C	
M		2 + 3 C (Figure 4)
M		2 + 3 C
M		2 + 3 C
M		3 + 3 *p* (Figure 7)
F		2 + 2 *p*
F		2 + 3 C (Figure 5)
F		2 + 3 C, 2 *p*
F		2 + 3 C
F		3 + 3 *p*
